# Preclinical and clinical study on [^18^F]DRKXH1: a novel β-amyloid PET tracer for Alzheimer’s disease

**DOI:** 10.1007/s00259-021-05421-0

**Published:** 2021-07-22

**Authors:** MiaoMiao Xu, Jun Guo, JiaCheng Gu, LinLin Zhang, ZiHao Liu, Lin Ding, HongLiang Fu, YuFei Ma, Sheng Liang, Hui Wang

**Affiliations:** 1 Department of Nuclear Medicine, Xinhua Hospital, School of Medicine, Shanghai Jiao Tong University, No. 1665, Kongjiang Street, Yangpu District, Shanghai, 200092 China; 2 Brain Injury Center, Department of Neurosurgery, Renji Hospital, School of Medicine, Shanghai Jiao Tong University, Shanghai Institute of Head Trauma, Shanghai, 200127 China

**Keywords:** Alzheimer’s disease (AD), β-Amyloid, Amyloid imaging, Positron emission tomography (PET), [^18^F]DRKXH1

## Abstract

**Background:**

The deposition of β-amyloid (Aβ) in the brain is a biomarker of Alzheimer’s disease (AD). Highly sensitive Aβ positron emission tomography (PET) imaging plays an essential role in diagnosing and evaluating the therapeutic effects of AD.

**Aim:**

To synthesize a new Aβ tracer [^18^F]DRKXH1 (5-(4-(6-(2-[18]fluoroethoxy)ethoxy)imidazo[1,2-alpha]pyridin-2-yl)phenyl) and evaluate the tracer performance by biodistribution analysis, in vivo small-animal PET-CT dynamic scan, ex vivo and in vitro autoradiography, and PET in human subjects.

**Methods:**

[^18^F]DRKXH1 was synthesized automatically by the GE FN module. Log *D* (pH 7.4) and biodistribution of [^18^F]DRKXH1 were investigated. Small-animal-PET was used for [^18^F]DRKXH1 and [^18^F]AV45 imaging study in AD transgenic mice (APPswe/PSEN1dE9) and age-matched normal mice. The distribution volume ratios (DVR) and standardized uptake value ratios (SUVRs) were calculated with the cerebellum as the reference region. The deposition of Aβ plaques in the brain of AD transgenic mice was determined by ex vivo autoradiography and immunohistochemistry. In vitro autoradiography was performed in the postmortem brain sections of AD patients and healthy controls. Two healthy control subjects and one AD patient was subjected to in vivo PET study using [^18^F]DRKXH1.

**Results:**

The yield of [^18^F]DRKXH1 was 40%, and the specific activity was 156.64 ± 11.55 GBq/μmol. [^18^F]DRKXH1 was mainly excreted through the liver and kidney. The small-animal PET study showed high initial brain uptake and rapid washout of [^18^F]DRKXH1. The concentration of [^18^F]DRKXH1 was detected in the cortex and hippocampus of AD transgenic mice brain. The cortex DVR of AD transgenic mice was higher than that of WT mice (*P* < 0.0001). Moreover, the SUVRs of AD transgenic mice were higher than those of WT mice based on the 0–60-min dynamic scanning. In vitro autoradiography showed a significant concentration of tracer in the Aβ plaque-rich areas in the brain of AD transgenic mice. The DVR value of [^18^F]-DRKXH1 is higher than that of [^18^F]-AV45 (1.29 ± 0.05 vs. 1.05 ± 0.08; *t* = 5.33, *P* = 0.0003). Autoradiography of postmortem human brain sections showed [^18^F]DRKXH1-labeled Aβ plaques in the AD brain. The AD patients had high retention in cortical regions, while healthy control subjects had uniformly low radioactivity uptake.

**Conclusions:**

[^18^F]DRKXH1 is an Aβ tracer with high sensitivity in preclinical study and has the potential for in vivo detection of the human brain.

**Supplementary Information:**

The online version contains supplementary material available at 10.1007/s00259-021-05421-0.

## Introduction

Alzheimer’s disease (AD) is the leading cause of dementia in elderly people worldwide, which emerges as one of the significant challenges to the health care system in the twenty-first century [1].

The typical pathophysiological changes of AD are the deposition of extracellular neuroinflammatory plaques and nerve fiber tangles in neurons. The deposition of β-amyloid (Aβ) plaques and phosphorylated tau protein results in neuronal damage and brain function damage. Nevertheless, the only gold standard for the diagnosis of AD is a detailed brain autopsy on the patient’s brain [2]. Nowadays, the main clinical diagnostic methods of AD include cognition and behavioral evaluation. When clinical symptoms, such as cognitive decline, occur, neurons are damaged irreversibly, and the potential repair opportunity is missed [3]. Therefore, sensitive and non-invasive detection of pathophysiological biomarkers is needed to facilitate early diagnosis, which is essential for the intervention treatments and delaying the development of AD. Aβ accumulation is an early event of AD and is widely considered the initial trigger for a cascade of other pathophysiological events [4]. Significantly advancing the diagnosis time of AD, in vivo imaging of Aβ plaques in the brain based on positron emission tomography (PET) plays an essential role in the pathophysiological mechanisms underlying AD development and the efficacy evaluation of the Aβ drug therapy [5].

In the past 20 years, a series of Aβ radioactive tracers, such as benzothiazole derivatives [^11^C]PIB (2-[4-(methyl-[^11^C]-amino) phenyl]-6-hydroxybenzothiazole) [6, 7], [^11^C]AZD2184(2-[6-(methylamino) pyridin-3-yl]-1,3-benzothiazol-6-ol), have been developed to visualize the deposition of Aβ plaques in the brain [8]. Although [^11^C]PIB is the most intensively studied tracer, its short half-life limits the clinical application. ^18^F-fluorination tracer has a suitable half-life, which enables the applicability of PET in a wide patient population. [^18^F]Flutemetamol(2-(3-[^18^F]4-(methylamino)phenyl)-1,3-benzothiazole-6-ol) [9] and [^18^F]AV45 ((E)-4-(2-(6-(2-(2-(2-[^18^F]fluoroethoxy)ethoxy)ethoxy)pyridine-3-yl)vinyl)-N-methyl benzenamine) [10] have been approved for clinical use. Nevertheless, these agents can detect Aβ plaques in AD patients’ brains; however, the relatively high retention of white matter decreases the signal-background ratio [8]. Moreover, these tracers cannot detect toxic amyloid oligomers, which contain neurotoxic substances and appear earlier than Aβ plaques.

Therefore, it is crucial to develop a new type of Aβ radioactive tracer to resolve these issues. [^125^I]DRK092 is an imidazopyridine single photoemission computed tomography (SPECT) probe with a high affinity to Aβ. Chen et al. reported that the binding power of [^125^I]DRK092 to Aβ was significantly higher than that of [^125^I]IMPY (2-(49-dimethylaminophenyl)-6-iodoimidazo[1,2-a]pyridine) [11, 12], with a high brain uptake rate and low non-specific binding. Positron emission tomography-computed tomography (PET-CT) is advantageous in diagnosing nervous system diseases because it has a higher resolution than SPECT. However, this restricts the application of [^125^I]DRK092 in PET-CT [13].

Thus, we modified the structure of DRK092 to improve its pharmacokinetics and facilitate [^18^F] labeling. In this study, we synthesized the new compound [^18^F]DRKXH1 and investigated a fully automated synthesis method of [^18^F]DRKXH1, which would be beneficial for future application. Herein, we evaluated the tracer performance by biodistribution analysis, in vivo small-animal PET-CT dynamic scan, ex vivo and in vitro autoradiography, and PET in human subjects.

## Materials and methods

### Animals

C57BL/6 mice were purchased from Shanghai SLAC Laboratory Animal Co., Ltd. AD transgenic mice (Genotype: APPswe/PSEN1dE9) were used in this study. The present study was approved by the Laboratory Animal Ethics and Welfare Committee Xinhua Hospital Affiliated to Shanghai Jiao Tong University School of Medicine (approval no. XHEC-F-2019–062). The Experimental Committee on Animal Ethics of Xinhua Hospital guidelines were followed for the care and use of animals. All animal experiments followed the animal welfare committee recommendations.

### Radiosynthesis of [^18^F]DRKXH1

The precursors and standard materials were purchased from WuXi AppTec Co. Ltd (Qidong, China). The reagents used in this study were commercial products and did not need further purification. The [^18^F]-fluoride produced by the accelerator was transferred to the module, dehydrated, and placed in the reaction tube, as described previously [14]. The precursor (3 mg) was dissolved in the dimethylformamide solution and heated at 120 °C for 20 min. Then, 2 mL saline was added to the reaction tube and subjected to high-performance liquid chromatography. The distillate was collected after 13 min and evaporated using a rotary evaporator for 10 min at 180 °C. Next, 5 mL saline was injected into the evaporator. The evaporator’s solution was filtered through a 0.22 μm sterile membrane (Millex-GV, Millipore). HPLC separation conditions: HPLC Column, XBridge C18, 10 × 250 mm, 10 μm (Waters, Milford, MA); mobile phase: 0.1% TFA + MeCN; flow rate 6 ml/min; conditions: 0–3 min 5%; 3–10 min 5–25%; 10–25 min 25–40%; 25–35 min 40%; 35–40 min 40–90%; 40–45 min 90%. The purified solution was identified by analytical HPLC. HPLC condition: Column, XBridge C18, 4.6 × 250 mm, 10 μm (Waters, Milford, MA), mobile phase: 45%: 55% acetonitrile: water, flow rate: 1 ml/min, UV: 350 nm. The purified solution was mixed with [19F]DRKXH1 standard and injected into HPLC system to identify whether the product in the solution was [^18^F]DRKXH1. [^18^F]-AV45 was radiolabeled according to a previously reported procedure [15].

### Lipophilicity measurement

According to the liquid–liquid partition method (shaking flask method), Log D_7.4_ of [^18^F]DRKXH1 was measured by partition between n-octanol and sodium phosphate buffer (pH 7.4) at room temperature [16].

### Biodistribution

A total of 30 wild-type (WT) mice (8 weeks old, female, 21–23 g) were used for the biodistribution study. [^18^F]DRKXH1 was injected into the tail vein. Then, the mice were euthanized by inhaling 100% carbon dioxide at the indicated time points of 1, 5, 15, 30, 45, and 60 min, and the group of each time point consisted of 5 mice. Subsequently, the mice were dissected, and the blood, whole brain, heart, liver, small intestine, spleen, lung, kidney, rectus femoris muscle, and femur were excised for weighed radioactivity count by a gamma counter (SN-695, Solar Ring Photoelectric Instrument, Shanghai, China). The enumeration data were converted into the percentage of the injected dose per gram of wet issue (% ID/g).

### In vivo small-animal-PETCT dynamic scan and MRI

In this PET-CT study, 22-month-old AD transgenic mice (female, *n* = 6) and age-matched WT mice (female, *n* = 5) were enrolled. The PET imaging used small-animal PET-CT (Siemens Medical Solutions, Knoxville, TN, USA). The scanning was started immediately after injection of [^18^F]DRKXH1 (17.46 ± 0.76 MBq) or [^18^F]-AV45 (17.53 ± 0.64) into mice with a tail vein indwelling needle. The dynamic scanning lasted for 60 min, and the acquisition was divided into 29 frames (12 × 10 s, 6 × 30 s, and 11 × 300 s). The PET scan data were modeled according to the protocols reported previously [17].

Mice were put in a radiofrequency coil and placed in an 11.7 T Bruker BioSpec high-field MRI system (Bruker BioSpin MRI GmbH, Ettlingen, Germany). Based on the MRI template images, we outlined cortex, cerebellum, whole brain, hippocampus, and petrous bone as the regions of interest (ROIs) to generate time-activity curves (TACs). The cerebellum was selected as the reference region, and the cortex was selected as the Aβ-rich region for analysis. A simplified reference Logan model was used instead of the plasma input function to calculate the distribution volume ratios (DVR) based on the 0–60-min dynamic PET scan [18]. On the other hand, standardized uptake value ratios (SUVRs) were calculated relative to the cerebellum for the entire dynamic PET scan.

The 22-month-old AD transgenic mice (female, *n* = 3) were assessed by dynamic PET scan for displacement studies. For this, [^19^F]DRKXH1 (10 mg/kg) was injected 40 min after [^18^F]DRKXH1 (18.15 ± 1.33 MBq) injection [19], and the whole brain was drawn as ROI to obtain the TAC during the 0–60 min dynamic.

### Ex vivo autoradiography

The 22-month-old AD transgenic mice (female, *n* = 6) and age-matched WT mice (female, *n* = 6) were injected with [^18^F]DRKXH1 (18.94 ± 0.88 MBq) into the tail vein. The animals were euthanized at 30, 40, and 60 min; then, the brain was removed immediately and sliced into 100-μm-thick sections. These sections were placed on an imaging plate (BAS-MS 2025, FUJIFILM, Japan) and exposed for 4 h, followed by scanning with Amersham™ Typhoon™ Biomolecular Imager (GE Healthcare) and the autoradiograms were analyzed by using ImageQuant TL 8.1 software (GE Healthcare). The cortex and cerebellum were drawn as ROIs.

### In vitro autoradiography

The paraffin brain Sects. (30 μm) of AD patients, healthy control subjects, and 22-month-old AD transgenic mice were incubated with [^18^F]DRKXH1 (0.3 nM) in 50 mM Tris–HCl buffer containing 40% ethanol for 1 h at room temperature [10]. [^19^F]DRKXH1 (10 μm) was incubated with [^18^F]DRKXH1 for the inhibition experiments [20]. Then, the sections were washed with saturated Li_2_CO_3_ in 40% ethanol, dipped into 40% ethanol for 2 min, and rinsed under flowing water for 30 s[10]. After drying, the brain sections were placed on an imaging plate (BAS-MS 2025, FUJIFILM, Japan) and exposed for 4 h, followed by scanning with Amersham™ Typhoon™ Biomolecular Imager (GE Healthcare).

### Immunohistochemical staining

The immunostaining was performed using Aβ1-42 (1:250 dilution) antibody (the detailed protocols are provided in the supplementary information). The human brain sections were stained for thioflavin-S, according to the methods described previously [21].

### PET study in human subjects

Based on the preclinical findings, we enrolled AD patients and healthy control subjects in performing a PET study after the injection of [^18^F]DRKXH1. The present study aimed to evaluate the effectiveness of [^18^F]DRKXH1 in distinguishing AD patients from healthy individuals. This study complied with the Declaration of Helsinki and was approved by the Ethics Committee Xinhua Hospital Affiliated to Shanghai Jiao Tong University School of Medicine (approval no. XHEC-F-2019–120). All subjects signed informed consent before participation in the study.

The AD patients were recruited, examined, and diagnosed by qualified attending neurologists. All physicians abided by the uniform diagnostic criteria, including detailed medical history and mini-mental state examination (MMSE).

All subjects underwent a PET assessment at 45 min after intravenous bolus injection of [^18^F]DRKXH1(370 MBq). Siemens Biograph 64PET/CT (Erlangen, Germany) was used for three-dimensional (3D) scanning. Before PET scanning, a low dose CT transmission scan was performed, and attenuation correction was performed. The image reconstruction was carried out by ordered subset expectation maximization 3D (OSEM 3D) method.

### Statistical analyses

All statistical analyses were performed using an IBM Statistical Package for Social Sciences. (SPSS) 22.0. All quantitative data were presented as mean ± standard deviation (SD) and compared using a two-sample Student’s *t* test. Statistical significance was set at *P* < 0.05.

## Results

### Radiosynthesis of [^18^F]DRKXH1

**Fig. 1:**
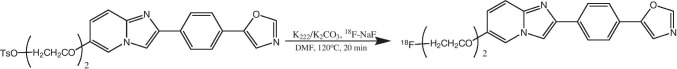
Radiosynthesis of [^18^F]DRKXH1

The multifunctional module automated synthesis of [^18^F]DRKXH1 required approximately 60 min, with a yield after decay correction of 40% and specific activity of 156.64 ± 11.55 GBq/μmol. The reaction equation is shown in Fig. 1. [^18^F]AV45 automated synthesis required approximately 50 min, with a yield after decay correction of 43% and specific activity of 176.43 ± 6.75 GBq/μmol.

### Lipophilicity measurement

The lipid-water distribution coefficient evaluated the lipophilicity of the probe and predicted the ability of the tracer to pass through the blood–brain barrier. Typically, the optimum log *D* (pH 7.4) range of Aβ tracer is about 1.5–3.5 [12]. and that of [^18^F]DRKXH1 measured by the liquid–liquid partition method (shaking flask method) was 2.78 ± 0.09 (*n* = 5).

### Biodistribution

**Fig. 2:**
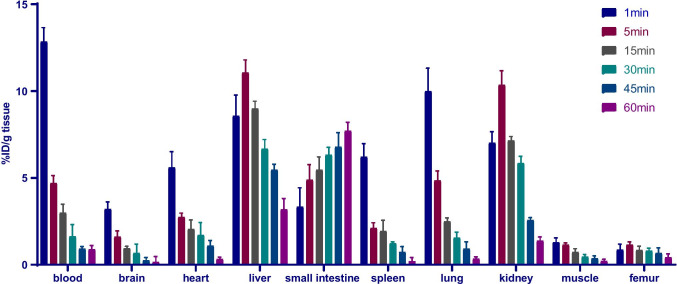
Biodistribution of [^18^F]DRKXH1 in WT mice at different time points. Values are expressed as %ID/g tissue (means ± SD, *n* = 5)

The biodistribution results revealed that the liver, kidney, and lung had a high uptake of [^18^F]DRKXH1 in the initial stage. The radioactivity in the lung, heart, and blood was rapidly cleared, while that in the liver and kidney was clarified slowly. The radioactivity in the small intestine increased gradually, which was similar to the previously reported distribution of Aβ imaging agents [22]. After injecting [^18^F]DRKXH1, the radioactivity of the femur was initially low, increased slightly at 5 min, and then remained stable (Fig. 2).

### In vivo small-animal-PETCT dynamic scan and MRI

**Fig. 3:**
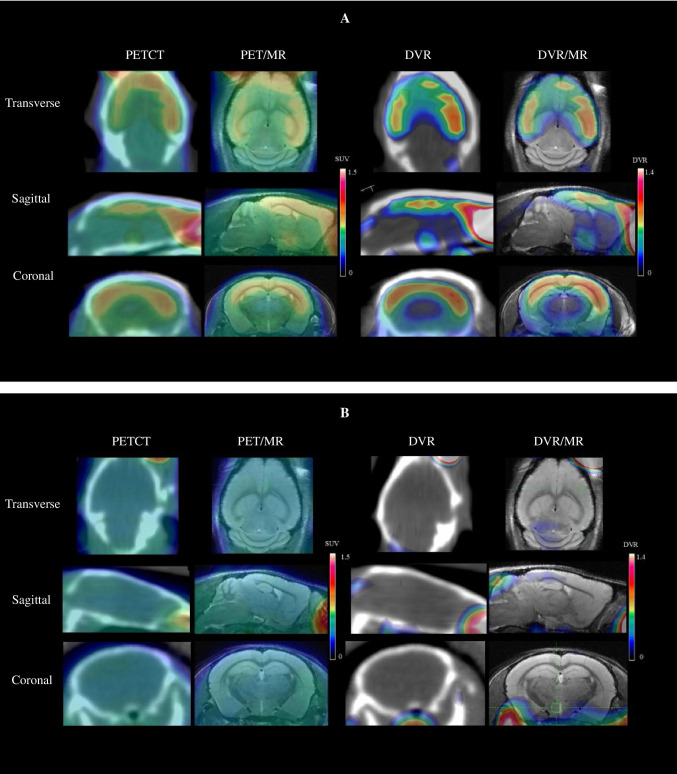

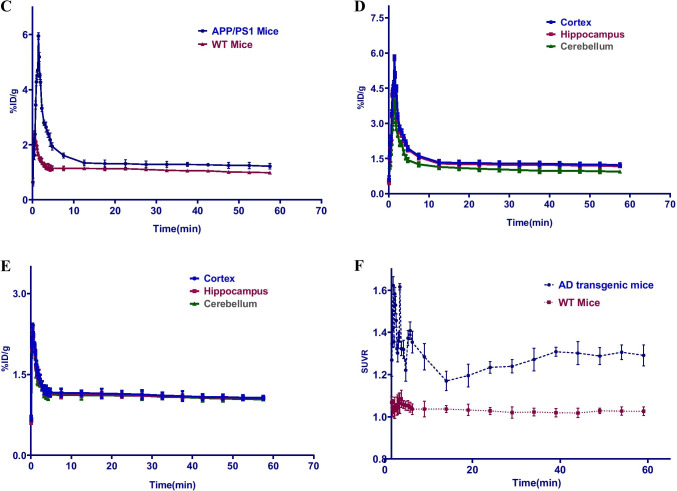

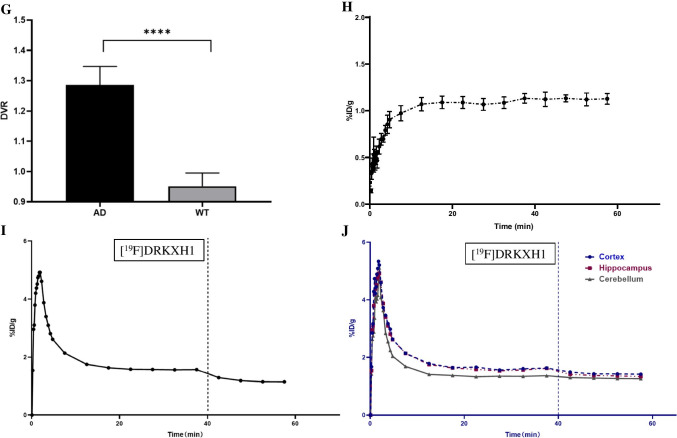
In vivo 0–60 min PET imaging with [18F]DRKXH1. **A** 22-month-old AD transgenic mice; **B** 22-month-old WT mice. **C** Whole brain 0–60 min time-activity curves (TACs) of the 22-month-old AD transgenic mice (*n* = 6) and age-matched WT mice (*n* = 5). The AD transgenic mice was outlined by the blue and WT mice was red line in the figure. **D** Cortex (blue), hippocampus (red), and cerebellum (green) time-activity curves (TACs) of the 22-month-old AD transgenic mice (*n* = 6) and **E** age-matched WT mice (*n* = 5). **G** [^18^F]DRKXH1 DVR (cortex/cerebellum) group comparisons for 22-month-old AD transgenic mice (*n* = 6) and age-matched WT mice (*n* = 5). ****P < 0.0001 (2-sample *t* test) (*P* < 0.0001, *t* = 10.35, mean: 1.29 vs 0.95); **F** [^18^F]DRKXH1 cortex-to-cerebellum SUVRs of 22-month-old AD transgenic mice and age-matched WT mice in 0–60 min dynamic scan. **H** Petrous bone 0–60 min time-activity curves (TACs) of the 22-month-old AD transgenic mice (*n* = 6). **I**, **J** Displacement study of [^18^F]DRKXH1 in AD transgenic mice. 0–60-min time-activity curves of whole brain, cortex, hippocampus, and cerebellum. Injected [^19^F]DRKXH1 at the 40th minute

In this study, the bodyweight of AD transgenic mice was significantly higher than that of WT mice (46.63 ± 1.71 g vs. 23.04 ± 1.13 g, *t* = 25.606, *P* < 0.001). In the imaging of 5–60-min frame fusion, we noticed a distinct radioactivity concentration in the cortex and other brain regions of AD transgenic mice. However, no specific radioactivity concentration was observed in the whole brain region of WT mice (Fig. 3A and B). The TACs of 0–60 min showed that the radioactivity uptake in the brain of AD transgenic and WT mice reached a peak at 2 min after injection of [^18^F]DRKXH1 and eluted rapidly (Fig. 3C). However, the tracer in the brain of AD transgenic mice was eluted more slowly than that of WT mice (Fig. 3C-E). Then, we fused the PET images with MR images and found that the radioactive concentration foci in the brain of AD transgenic mice were located in the cortex and hippocampus (Fig. 3A and B).

Next, we drew the cortex, hippocampus, and the cerebellum of AD transgenic mice as ROIs. Considering the cerebellum as the reference area, we obtained the SUVR of the cortex to the cerebellum and hippocampus to the cerebellum (Fig. 3F). The DVR value of AD transgenic mice was significantly higher than that of WT mice (1.29 ± 0.05 vs. 0.95 ± 0.04; *t* = 10.35, *P* < 0.0001) (Fig. 3G).

**Fig. 4:**
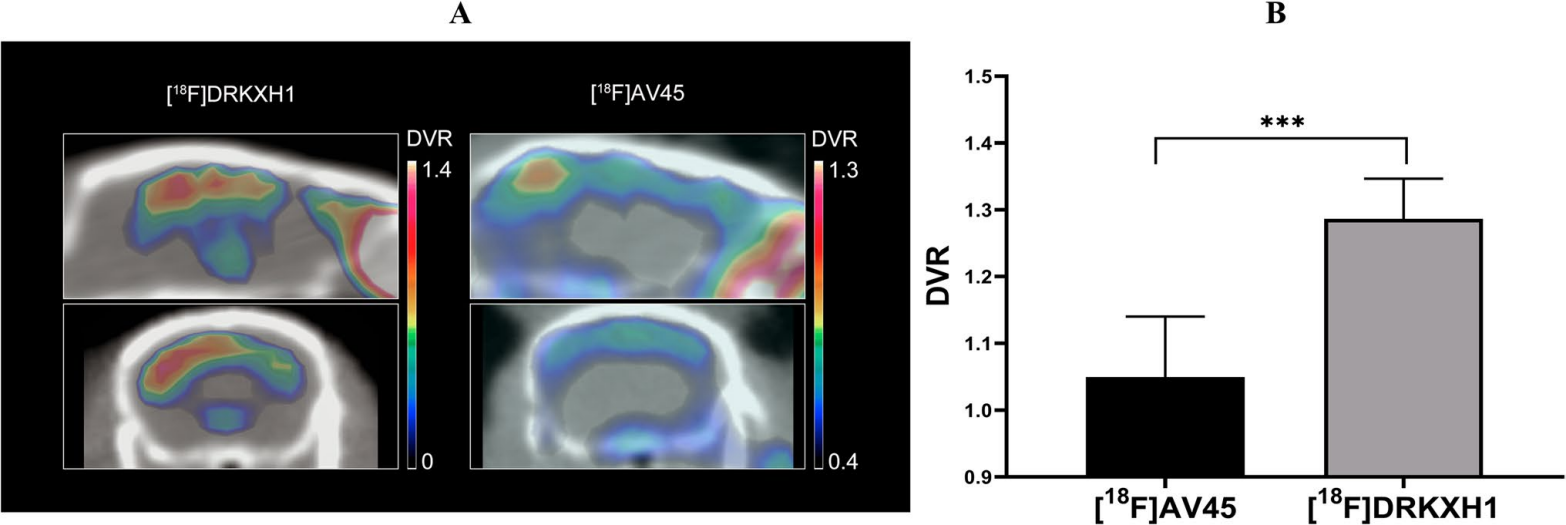
**A** In vivo 0–60 min PET imaging with [^18^F]DRKXH1 and [^18^F]AV45 in 22-month-old AD transgenic mice. **B** DVR (cortex/cerebellum) group comparisons for [^18^F]DRKXH1 and [^18^F]AV45 mice (*n* = 5). ***P < 0.0005 (2-sample *t* test, 1.29 ± 0.05 vs. 1.05 ± 0.08; *t* = 5.33, *P* = 0.0003)

To further evaluate the ability of [^18^F] DRKXH1 to detect Aβ plaques in the brain of AD transgenic mice, we directly compared the dynamic PETCT imaging results of [^18^F] DRKXH1 and [^18^F] AV45 in AD transgenic mice (Fig. 4A). The retention of [^18^F] DRKXH1 in the cerebellum is less than that of [^18^F]AV45 (Supplements Fig. 1). The clearance rates of the two tracers in the brain of AD transgenic mice were similar (Supplements Fig. 1). The DVR value of [^18^F] DRKXH1 was higher than that of [^18^F]-AV45 (1.29 ± 0.05 vs. 1.05 ± 0.08; *t* = 5.33, *P* = 0.0003) (Fig. 4B).

In the 60-min dynamic scan, the TAC of petrous bone showed that radioactivity increased at the initial stage (0–5 min) but remained stable subsequently (Fig. 3H).

Next, we conducted displacement experiments to evaluate the specificity of [^18^F]DRKXH1 binding to Aβ plaques in the brain of AD transgenic mice. Imaging results showed that at 40–60 min, the TAC curves of mouse whole brains were decreased but not significantly (Fig. 3I, J).

### Ex vivo autoradiography

**Fig. 5:**
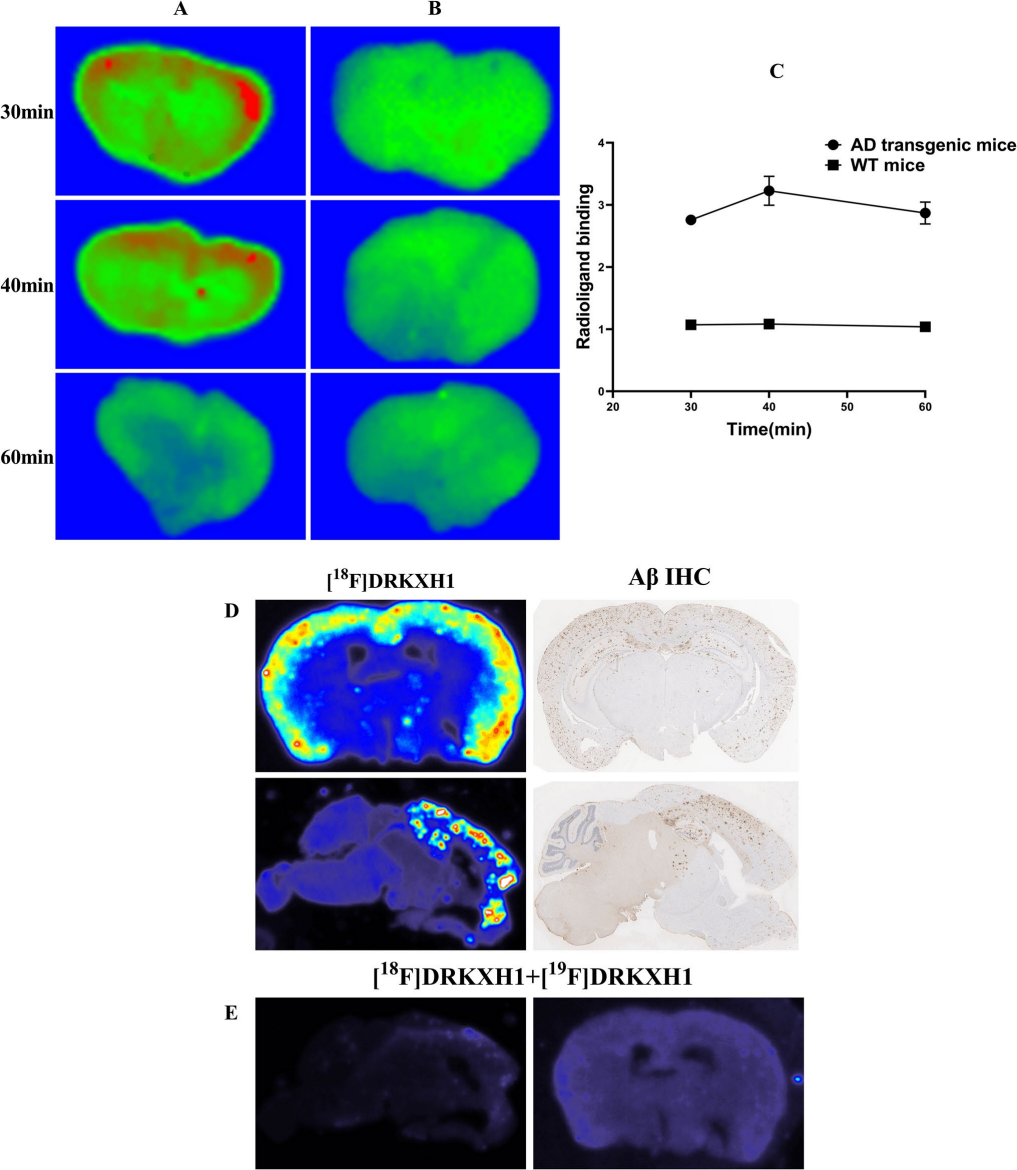
Ex vivo autoradiography. **A** AD transgenic mice; **B** WT mice; **C** radioligand binding was quantified as ratios of radioactivity intensity of cortex to cerebellum. **D** In vitro autoradiography of AD transgenic mice and immunohistochemical staining of Aβ plaques of AD transgenic mice; **E** in vitro autoradiography using [^18^F]DRKXH1 + 10 μM [^19^F]DRKXH1of AD transgenic mice

We used ex vivo autoradiography to verify that [^18^F]DRKXH1 binds to Aβ plaques in the brain of AD transgenic mice. After 30 and 40 min of the imaging agent injection, significant radioactivity concentration was detected in the cortex of AD transgenic mice, but no specific radioactivity concentration was observed in the whole brain region of WT mice (Fig. 5A and B). Radioligand binding was quantified as ratios of radioactivity intensity of cortex to cerebellum (Fig. 5C).

### In vitro autoradiography

**Fig. 6:**
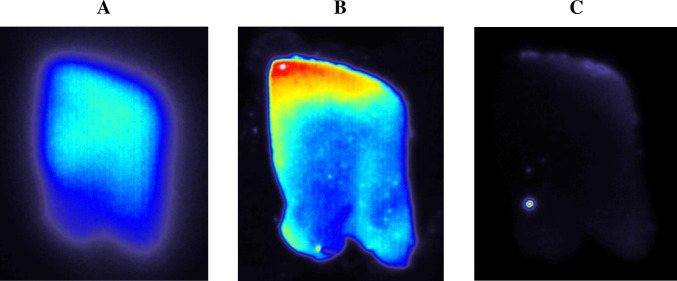
In vitro autoradiography of human brain sections labeled with [^18^F]-DRKXH1. **A** Healthy control subjects. **B** AD patient. **C** In vitro autoradiography using [^18^F]DRKXH1 + 10 μM [^19^F]DRKXH1 of AD patient

The brain sections of AD transgenic mice showed dense labeling of the plaques in the cortex and hippocampus regions (Fig. 5C). The autoradiography showed distinct deposition of [^18^F]DRKXH1 in the human cortex which enriched Aβ plaques, and there was no specific concentration in a healthy human brain (Fig. 6B). After co-incubation with [^19^F] DRKXH1, the radioactivity of the brain sections of mice and human showed a dramatic decrease (Fig. 5E and Fig. 6C).

### IHC and thiosemicarbazone S staining

The positive result of diaminobenzidine staining was brown granules. Aβ1-42 IHC showed that the AD transgenic mice cortex and hippocampus had abundant Aβ plaques, while no plaque deposition was observed in the cerebellum (Fig. 5D). Thiosemicarbazone S staining results showed Aβ deposition in human brain (supplement).

### PET study in human subjects

**Fig. 7:**
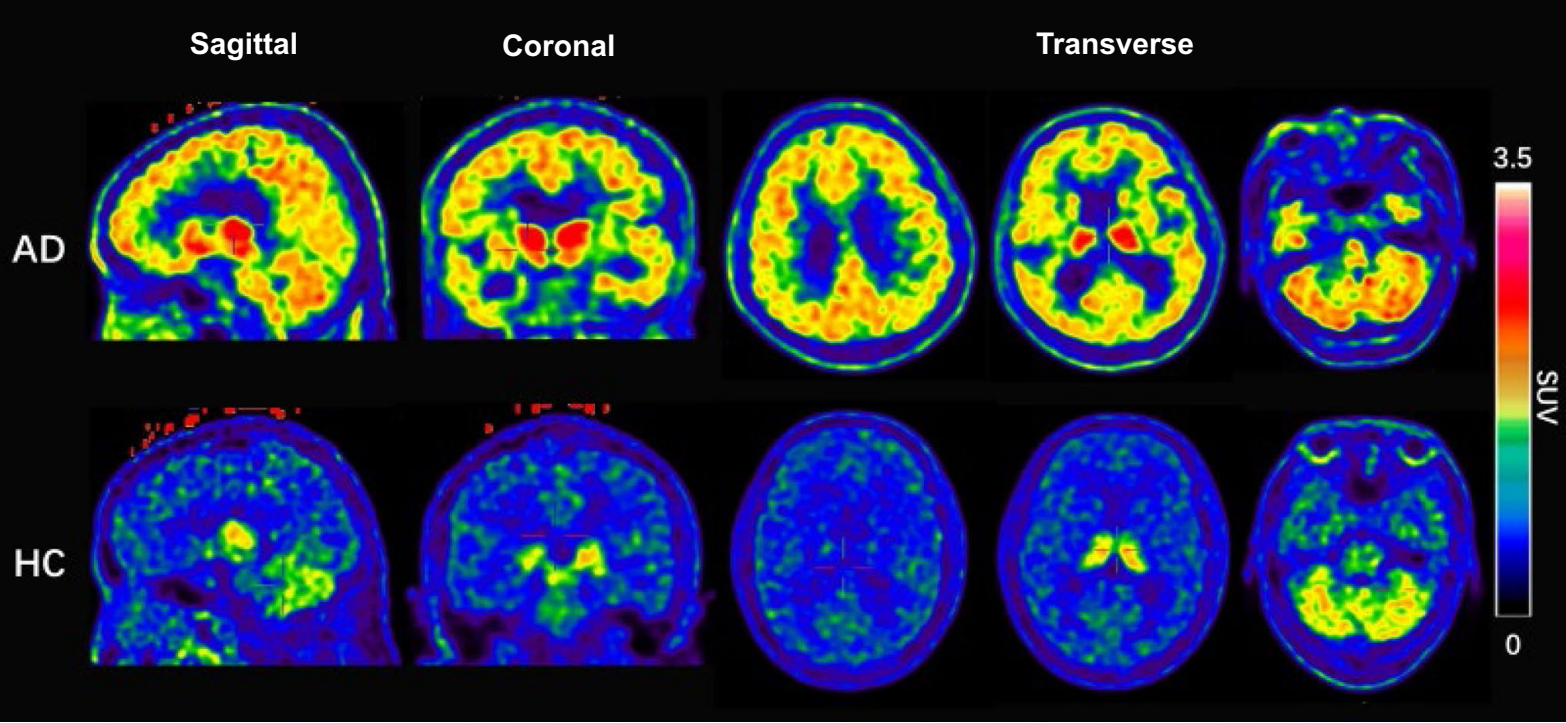
[^18^F]-DRKXH1 PET images of an AD patient and a healthy control subject

In this study, we enrolled 1 AD patient (male, 82 years, MMSE: 17) and 2 healthy control subjects (male, 42 and 50 years old, respectively). In AD patients, we observed significant [^18^F]DRKXH1 retention in recognized amyloid deposit areas, such as the parietal and frontal cortex. However, this phenomenon was not observed in healthy control subjects. In both groups, we observed low retention of subcortical white matter and high uptake in the cerebellar cortex, pons, and thalamus (Fig. 7 and Supplement Table 1).

## Discussion

The radiochemical labeling of [18F]DRKXH1 adopts a nucleophilic [^18^F] substitution reaction, which is synthesized by a one-step method. The labeling process is simple, the automatic synthesis procedure is mature, and the specific activity of [18F]DRKXH1 is high, which meets the criteria of neuroimaging agents for clinical applications.

In PET imaging study, [^18^F]DRKXH1 is a small molecule and moderate lipophilic tracer. [^18^F]DRKXH1 shows potential for ideal Aβ PET tracer, indicating that it can penetrate the complete blood–brain barrier and has high initial uptake in the frontal brain of mice[12]. In AD transgenic mice, the brain uptake peaked at 6%ID/g. We observed that the initial brain uptake in AD transgenic mice was higher than that in WT mice. In addition, the bodyweight of AD transgenic mice was significantly higher than that of WT mice (46.63 ± 1.71 g vs. 23.04 ± 1.13 g, *t* = 25.606, *P* < 0.001), which is consistent with previous reports, wherein high initial brain uptake was detected in high-weight mice [23]. Studies have showed that AD is related to BBB damage, which may lead to increased permeability [24–26]. Therefore, we speculate that this phenomenon may be explained by BBB damage. Moreover, the presence of Aβ in AD transgenic mice, serving as the target site on [^18^F]DRKXH1, may also cause the higher initial brain uptake in AD transgenic mice. Also, after the injection of [^18^F]DRKXH1, AD transgenic mice had significantly higher DVR values than WT mice, indicating that [^18^F]DRKXH1 has a high retention rate in the Aβ-rich brain regions and can locate Aβ plaques in the brain. The shallow non-specific binding provides high signal-to-noise images beneficial to the diagnosis of AD and efficacy evaluation of therapies targeting Aβ. Intriguingly, rodents are susceptible to the defluorination of many [^18^F] labeled radioactive tracers, leading to a gradual increase in radioactivity in the skeleton [22, 27]. The radioactive spillover from the skull introduced considerable error into the brain regions, as quantified by PET scans [28]. Also, we observed a mild increase in bone radioactivity 0–5 min after injection, which was subsequently stable without apparent detachment during 5–60 min (0.97–1.1% ID/g). [^18^F]-AV45 is an Aβ imaging agent approved by the FDA. Similar to [^18^F]-AV45, [^18^F]-DRKXH1 exhibits uptake that closely mirrored Aβ deposition in the brain, with higher DVR values than [^18^F]-AV45. However, the difference in the autoradiography of human brain sections proposed that [^18^F]DRKXH1 can distinguish between AD patients and healthy controls. Thus, this study verified the binding ability of [^18^F]DRKXH1 to Aβ, but the experimental verification of the binding power of toxic amyloid oligomers is yet lacking. Next, we plan to perform PET imaging on transgenic mice for longitudinal studies using brain homogenates from these mice of different ages, Aβ fibers in human brain homogenates, and synthetic human Aβ fibers. The binding affinity assay verified the binding ability of DRKXH1 to Aβ oligomers. The results of preclinical experiments showed that [^18^F]DRKXH1 has a high binding affinity and excellent imaging potential for Aβ in the brain of AD transgenic mice, low non-specific binding, and is rapidly eluted from the healthy brain.

The initial clinical study results showed noticeable retention of [^18^F]DRKXH1 in the cortex of AD patients, which did not appear in healthy subjects, indicating the efficiency of the compound in distinguishing between AD patients and healthy individuals. Low subcortical white matter retention was observed in both groups of individuals; however, no significant boundary existed between cortex and white matter in patients with obvious brain atrophy. Considering the false-positive results caused by high non-specific uptake of white matter, low uptake of white matter might contribute to the accuracy and micro detection capability of Aβ deposition. Conversely, the cerebellar cortex, pons, and thalamus also showed high non-specific uptake of [^18^F]DRKXH1 in both groups. The lack of mechanism explanation and non-specific uptake of the cerebellar cortex might pose challenges to the quantitative analysis of the imaging results. Taken together, the current evaluation results constitute the preliminary imaging report of [^18^F]DRKXH1, necessitating additional subjects for the kinetic study. The follow-up data might contribute to the further characteristic analysis of [^18^F]DRKXH1 and the suitable selection of reference region for quantitative analysis.

Collectively, [^18^F]DRKXH1 is an Aβ tracer with high sensitivity in preclinical study, and has the potential for in vivo detection of the human brain.

## Conclusion

In this study, we synthesized a new Aβ tracer [^18^F]DRKXH1. It showed a high binding affinity at in vivo and in vitro level in the preclinical study. The PET study in human subjects indicated its potential in clinical diagnosis but further research is still needed.

## Limitations

This study has some limitations. This study did not use the classical PET tracer such as [^11^C]PIB and [^18^F]florbetapir for imaging comparison. We did not use [^18^F]DRKXH1 to longitudinally monitor Aβ plaques deposition in the brain of transgenic mice.

## Supplementary Information

Below is the link to the electronic supplementary material.

Supplementary file1 (PDF 553 KB)
