# Supplementary material for: Preclinical and clinical study on [18F]DRKXH1: a novel β-amyloid PET tracer for Alzheimer’s disease
Source: Eur J Nucl Med Mol Imaging. 2021 Jul 22;49(2):652–63. doi: 10.1007/s00259-021-05421-0 (PMC8803783; doi:10.1007/s00259-021-05421-0)
Supplement: Supplementary file 1 — Supplementary file1 (PDF 553 KB) [file 259_2021_5421_MOESM1_ESM.pdf]

MATERIALS AND METHODS

1.Immunohistochemical staining

Paraffin-embedded sections of mouse brain tissue (Leica, RM2235) successively put the slices into xylene I 15min-xylene II 15min-anhydrous ethanol I 5min-anhydrous ethanol II 5min Mel 85% alcohol 5min Mel 75% alcohol 5min-distilled water wash. The tissue sections were placed in a repair box filled with EDTA antigen repair buffer (PH8.0) for antigen repair in the microwave oven, 8min to boil, cease-fire 8min heat preservation, and then turn to medium-low heat for 7 minutes. During this process, excessive evaporation of the buffer should be prevented, and no dry tablets should be taken. After natural cooling, the glass slides were placed in PBS (PH7.4) and shaken and washed on the decolorizing shaker for three times, each time 5min. The slices were incubated in 3% hydrogen peroxide solution (hydrogen peroxide: pure water = 1:9) and incubated at room temperature for 25 min. The slides were placed in PBS (PH7.4) and shaken and washed on the decolorizing shaker three times, each time 5min. After the section was slightly dried, a histochemical pen was used to draw a circle around the tissue (to prevent the antibody from flowing away). The tissue was evenly covered with 3% BSA in the circle, and the 30min was sealed at room temperature. Gently get rid of the sealing solution, add BSA (Solarbio) to the slices, and the dilution ratio is 1： 1000A β 1-42 antibody (Abcam), slices are placed flat in the wet box at 4 °C to incubate overnight. The glass slides were placed in PBS (PH7.4) and shaken and washed on the decolorizing shaker for three times, each time 5min. After slightly drying, the slices were dripped with secondary antibody HRP-goat anti-rabbit (KPL) covering tissue in the circle, 50min was incubated. The glass slides were placed in PBS (PH7.4) and shaken and washed on the decolorizing shaker for three times, each time 5min. After the slices were slightly dried, the freshly prepared DAB chromogenic solution was dripped in the circle. The coloration time was controlled under the microscope, the positive was brown, and the slices were rinsed with tap water to stop coloring. Harris hematoxylin re-staining 3min, tap water washing, 1% hydrochloric acid alcohol differentiation for a few seconds, tap water rinse, ammonia return blue, running water rinse. Put the slices in 75% alcohol 6min, 85% alcohol, 6min, anhydrous ethanol I 6min, anhydrous ethanol II 6min, xylene I 5min, dehydration and transparent, take out the slices from xylene to dry slightly and seal the tablets with neutral gum. Microscopic examination of (NIKON ECLIPSE CI-S), image acquisition and analysis (Nikon DS-U3).

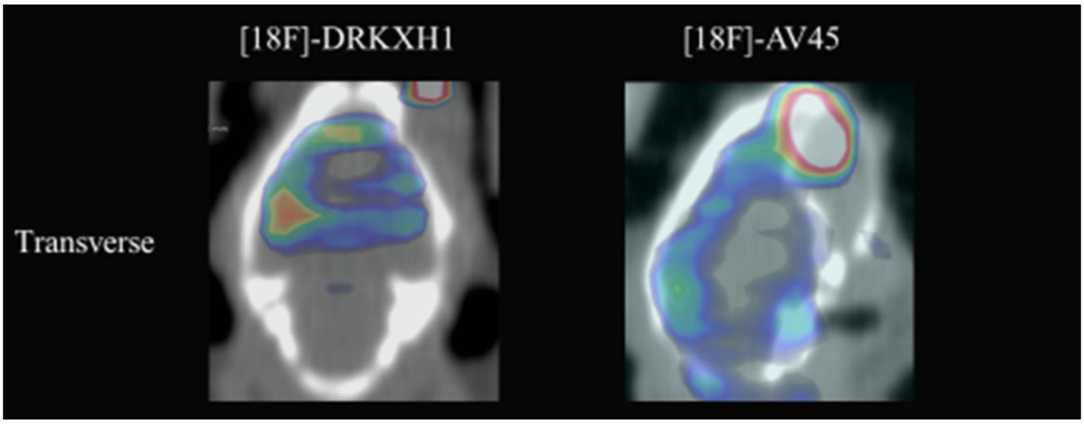

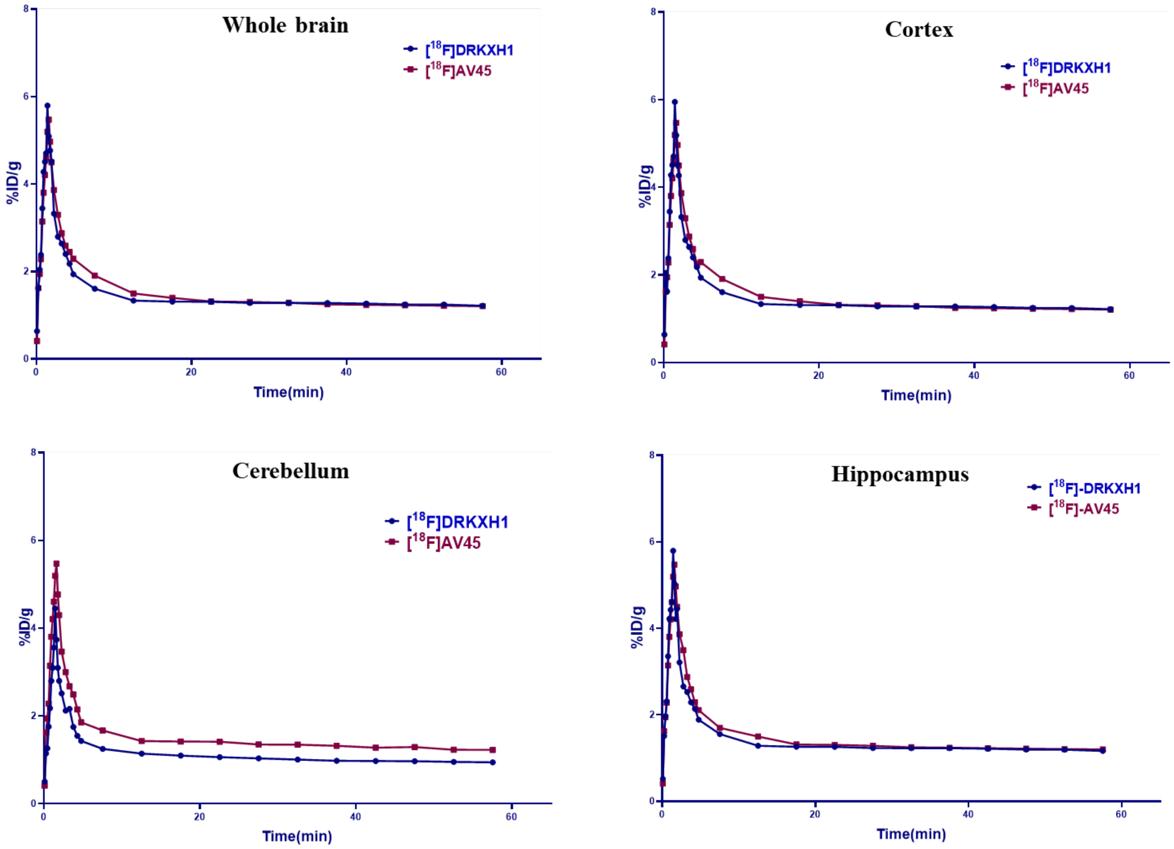

Supplemental figure 1. In vivo 0-60 minutes PET imaging and cortex, whole brain, cerebellum , hippocampus TACs with [^{18}F]DRKXH1 and [^{18}F]AV45 in 22month-old APP/PS1mice. (B) DVR (cortex/cerebellum) group comparisons for [^{18}F]DRKXH1 and [^{18}F]AV45 mice (n=5). ***P <0.0005 (2-sample t test, 1.29 ± 0.05 vs. 1.05 ± 0.08; t = 5.33, P = 0.0003).

A .

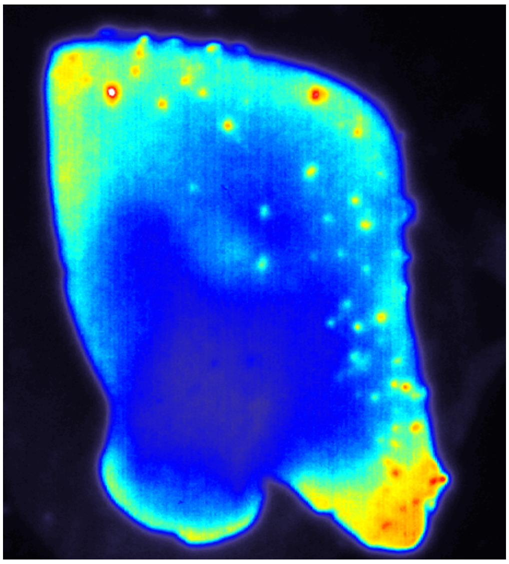

B.

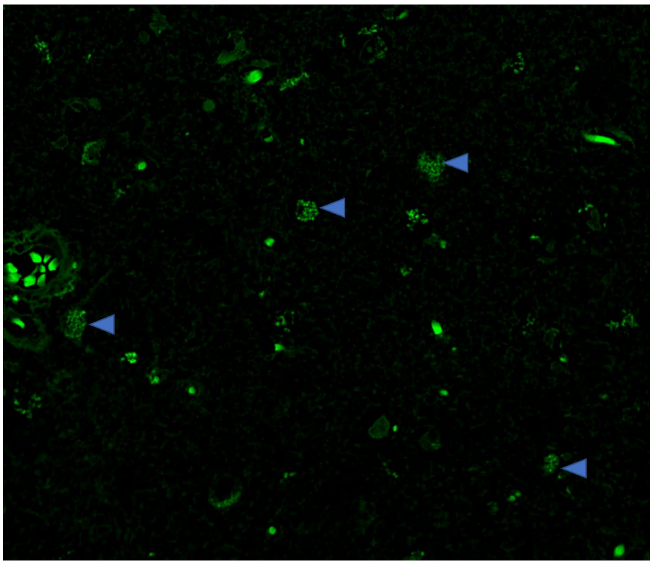

Supplemental figure 2. (A) In vitro autoradiograms of AD patient brain sections labeled with [^{18}F]DRKXH1 healthy control. (B). Thioflavin S staining of Aβ plaques in AD patient brain sections (blue arrows) Scale bars: 50 μm.

Supplemental table1. Regional standardized uptake value ratios (SUV)

Region	AD	HC
PCG	2.1	0.9
CER	2.4	1.9
SWM	1.1	0.7
PC	2.1	1.0
TC	1.8	1.0
OCC	1.6	1.0
OFC	2.1	1.0
PAR	2.2	1.0
PONS	1.9	1.3
THA	3.3	1.8

PCG posterior cingulate gyrus, CER cerebellum, SWM subcortical white matter, PC prefrontal cortex, TC temporal cortex, OCC occipital cortex, OFC orbitofrontal cortex, PAR parietal cortex, PONS pons, THA thalamus
